# A Nonlinear Suspension Road Roughness Recognition Method Based on NARX-PASCKF

**DOI:** 10.3390/s24216938

**Published:** 2024-10-29

**Authors:** Jiahao Qian, Yinong Li, Ling Zheng, Huan Wu, Yanlin Jin, Linhong Yu

**Affiliations:** 1 College of Mechanical and Vehicle Engineering, Chongqing University, Chongqing 400044, China; 202307131172t@stu.cqu.edu.cn (J.Q.); zling@cqu.edu.cn (L.Z.); 201934131007@cqu.edu.cn (H.W.); 202032021109t@cqu.edu.cn (Y.J.); 202307131311t@stu.cqu.edu.cn (L.Y.); 2 State Key Laboratory of Mechanical Transmission for Advanced Equipment, Chongqing University, Chongqing 400044, China

**Keywords:** road roughness identification, square root cubature Kalman filter, nonlinear auto-regressive with exogenous, adaptive Kalman filtering, nonlinear suspension

## Abstract

Road roughness significantly impacts vehicle safety and dynamic responses. For nonlinear suspension systems, the nonlinear characteristics often make it challenging for estimators to identify the actual road roughness accurately. This paper proposes a hybrid road roughness identification algorithm based on nonlinear auto-regressive with exogenous inputs (NARX) and a process noise adaptive square root cubature Kalman filter (PASCKF) to address this issue. Driven by vehicle acceleration data, an NARX-based road roughness identification system is constructed to mitigate the model uncertainties. Furthermore, a hybrid strategy is proposed. On the one hand, the accurate road roughness estimated by the NARX is converted into process noise covariance, enhancing the estimator’s accuracy and convergence rate. Another switching strategy is proposed to optimize the non-convergence issues of the PASCKF. Finally, simulation and actual vehicle experiment data demonstrate that this approach offers superior identification accuracy and adaptability compared to the standalone SCKF algorithm.

## 1. Introduction

With the widespread adoption of intelligent vehicles, automotive comfort and safety have garnered significant attention. Among the factors affecting these aspects, driving comfort is closely linked to road roughness. Road roughness refers to the degree of irregularity of the road, which serves as the primary source of excitation that induces vehicle body vibrations during driving [1]. The ability to quickly and accurately identify road surface roughness is essential for enhancing vehicle handling stability, improving comfort, and prolonging the lifespan of components. Currently, the methods for determining road surface roughness can be broadly categorized into three types: contact measurement methods, non-contact measurement methods [2], and road roughness identification based on vehicle responses [3,4,5].

The contact measurement method offers high accuracy at low to medium speeds. However, the requirement for specialized road surface measurement equipment incurs high costs and results in low measurement efficiency, which limits its applicability on a large scale [6]. In contrast, non-contact measurement methods that utilize onboard sensors such as LiDAR, ultrasonic radar, or cameras provide the advantages of minimal identification errors and robust real-time performance. Despite these benefits, such systems are complex and costly, necessitating regular maintenance and calibration. Furthermore, non-contact methods are sensitive to environmental conditions; adverse weather or poor lighting can significantly impact the measurement accuracy, thereby hindering their effectiveness in large-scale road surface detection [7]. To address these limitations, this paper investigates a method for estimating road roughness based on the vehicle dynamic responses obtained from sensor data.

Road roughness estimation methods based on vehicle dynamic responses can be categorized into two primary approaches: data-driven methods and model-based methods. Data-driven approaches establish a mapping between road roughness and vehicle dynamic responses without relying on a specific physical model. For instance, Qin et al. [8] proposed an adaptive neuro-fuzzy inference system (ANFIS) for road roughness identification, comparing its performance to recursive least squares (RLS) and the group method of data handling (GMDH) under various vehicle speeds and road excitations. Farshad et al. [9] introduced a road roughness estimation method based on artificial neural networks, utilizing data collected from multiple smartphones. In this approach, the vertical acceleration, position, and speed features were extracted to train the network, which outputs the International Roughness Index (IRI) for road grade classification. Similarly, Lee et al. [10] developed an artificial intelligence-based road grade recognition method by combining continuous wavelet transform with convolutional neural networks.

While these studies utilized traditional artificial neural networks, such models often feature simplistic architectures and fail to account for the temporal dependencies inherent in sequential data. As a result, their efficiency and accuracy are limited. Recurrent neural networks (RNNs), particularly those based on long short-term memory (LSTM) architectures, have shown superior performance in time series forecasting, making them more suitable for road profile estimation by leveraging long-term dependencies in the data. Li et al. [11] proposed a road roughness estimation framework using advanced RNN architectures, while Jiang et al. [12] trained an LSTM network with sprung and unsprung mass acceleration data, applying the model to identify road grades through the recognition of unsprung mass acceleration patterns. Furthermore, Guanqun Liang et al. [13] collected acceleration data from real-world roads to train an LSTM network, which was subsequently employed for road excitation identification, yielding promising results in real-vehicle experiments.

Similarly, model-based identification methods do not require external measuring devices, offering low cost and broad applicability. For example, Kattar et al. [14,15] proposed a closed-loop estimator for unknown input road classification, which estimates road roughness by analyzing the frequency characteristics of the transfer function and utilizing vehicle response data obtained from simulations, test benches, and real roads. Jiang et al. [16] employed a transfer function linking tire forces to road elevation to identify road roughness. Liu et al. [17] designed a road roughness observer based on suspension characteristics, using the sprung mass acceleration as the input for roughness identification and road classification through power spectral density analysis. Rana and Asaduzzaman [18] introduced an inverse road reconstruction method, collecting vertical acceleration data from the actual vehicle to formulate a new suspension dynamics state-space equation. Tan et al. [19] proposed a road classification method utilizing a Bayesian regression-based self-measurement algorithm, which employed the non-DC gain in the transfer function for road classification estimation. Zhang et al. [20] estimated road surface roughness using the actual full-vehicle response, deriving their method from the vehicle’s frequency response function (FRF) based on motion equations, and validated their road roughness estimation through real-vehicle experiments.

In the above research, many scholars focused on linear passive suspension systems, assuming constant stiffness and damping. However, with the ongoing development of semi-active and active suspensions, the parameters of these systems may vary. Furthermore, estimators based on passive suspensions are linear; when applied to nonlinear suspensions with variable stiffness and damping, these linear estimators face challenges in capturing nonlinear dynamic responses, leading to reduced estimation accuracy and, in some cases, divergence. Thus, there is a lack of road estimation techniques that account for variable spring stiffness and damping parameters.

To address the aforementioned issues, this paper proposes a road roughness estimation method that combines the nonlinear auto-regressive neural network with exogenous inputs (NARX) and the process noise adaptive square root cubature Kalman filter estimator (PASCKF). Firstly, this study utilizes the NARX to establish the relationship between vehicle acceleration time series and road roughness. Unlike traditional static neural networks, the NARX employs feedback and memory, allowing it to retain data from previous time steps and incorporate it into subsequent calculations. This dynamic characteristic enables the NARX neural network to capture more comprehensive system information, thereby improving the accuracy of the road roughness identification and prediction. Additionally, to effectively filter out noise and accurately estimate the dynamic response of nonlinear suspension systems, this paper introduces a process noise adaptive PASCKF. Finally, a fusion strategy is proposed: on the one hand, the accurate road roughness estimated by the NARX is converted into process noise covariance to enhance the accuracy and convergence speed of the PASCKF estimator; on the other hand, a switching strategy is introduced to address the non-convergence issues of the PASCKF.

This paper is dedicated to investigating the identification of road roughness across different surface levels while also considering a nonlinear suspension system with variable spring stiffness and damping coefficients. The rest of this paper is organized as follows. Section 2 establishes the random road model and nonlinear suspension model. Section 3 introduces the construction of the NARX network to apply vehicle response signal identification and designs the NARX network for road roughness. Section 4 designs the PASCKF estimator and proposes a hybrid strategy to estimate the road classification and road level. Section 5 conducts simulation experiments for single road surfaces and joint road surfaces. Section 6 conducts validation of the real-vehicle experiments. Section 7 presents the general conclusion.

## 2. System Model Establishment

### 2.1. Random Road Model

Under normal driving conditions, road roughness is the primary influencing factor of vehicle body vibration. To describe the impact of road roughness on vehicle vibration, the time-domain signal is constructed using the random filtering white noise method. Based on the power spectral density function, the time-domain signal of road unevenness is constructed below [21]:

(1) $${\dot{z}}_{r}(t)=-2\pi n_{c}vz_{r}(t)+2\pi n_{0}\sqrt{G_{q}(n_{0})v}w(t)$$

where, $z_{r}(t)$ represents the time-domain road unevenness, v denotes the vehicle speed, $n_{c}$ is the lower cutoff frequency of the road spatial frequency, taken as $n_{c}$ = 0.01 m^−1^, w(t) represents the random unit white noise, $G_{q}(n_{0})$ is the power spectral density of the road unevenness at the reference spatial frequency, with partial road classification levels shown in Table 1.

### 2.2. Description of Road Unevenness

To establish a connection between road grade and road roughness, this paper utilizes the International Roughness Index (IRI) for a description. The IRI is an internationally recognized metric for evaluating road quality, as proposed by the World Bank. It represents the cumulative absolute value of the suspension dynamic deflection per unit distance at a speed of 80 km/h. The calculation formula is as follows:

(2) $$IRI=\frac{1}{L}\int_{0}^{t}\left|{\dot{z}}_{s}-{\dot{z}}_{u}\right|dt$$

where *L* and *t* represent the total distance traveled by the vehicle and the total time, respectively.

### 2.3. Nonlinear Suspension Model

Considering that this paper investigates a nonlinear suspension system, a nonlinear spring and damping model has been established. In this context, the nonlinear characteristics of damping are considered, primarily because the damping force is asymmetric during compression and extension strokes. To analyze these nonlinear characteristics, this paper employs a polynomial fitting method to represent the velocity-damping force characteristic curve of the damper:

(3) $$F_{c}=c_{s}^{l}\left({\dot{z}}_{s}-{\dot{z}}_{u}\right)+c_{s}^{a}\left|{\dot{z}}_{s}-{\dot{z}}_{u}\right|+c_{s}^{n}\sqrt{\left|{\dot{z}}_{s}-{\dot{z}}_{u}\right|}\mathrm{sgn}\left({\dot{z}}_{s}-{\dot{z}}_{u}\right)$$

where $c_{s}^{l}$ represents the linear damping coefficient, $c_{s}^{n}$ denotes the nonlinear damping coefficient, and $c_{s}^{a}$ is the coefficient associated with the asymmetry of the damper.

Similarly, the nonlinearity of the spring is also taken into account:

(4) $$F_{k}=k_{s}^{l}\left(z_{s}-z_{u}\right)+k_{s}^{n}{\left(z_{s}-z_{u}\right)}^{3}$$

where $k_{s}^{l}$ represents the linear stiffness coefficient of the spring while $k_{s}^{n}$ denotes the nonlinear stiffness coefficient.

**Figure 1:**
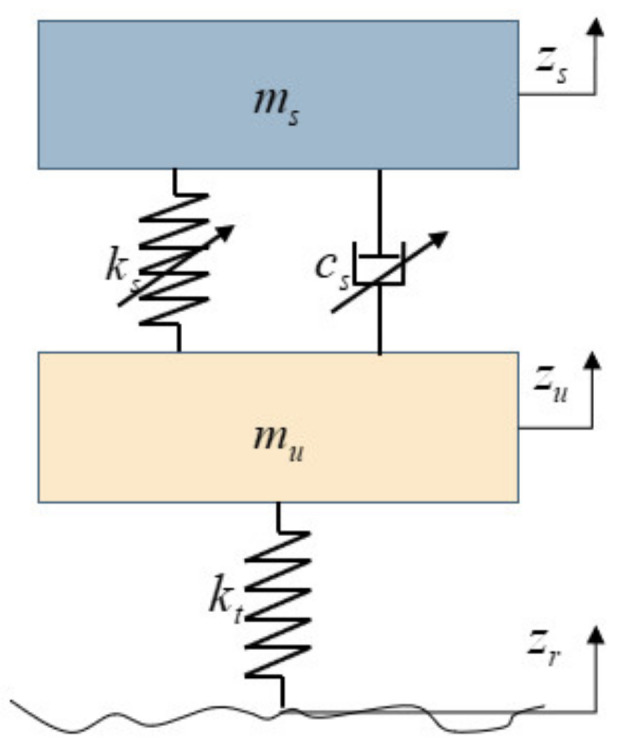
One-fourth nonlinear suspension model.

Therefore, the dynamic equations of the nonlinear vehicle model can be expressed as follows and the schematic diagram of suspension is illustrated in Figure 1.

According to Newton’s laws of motion, the one-fourth suspension function can be written as follows:

(5) $$\left\{\begin{array}{l} m_{s}{\ddot{z}}_{s}+F_{k}+F_{c}=0\\ m_{u}{\ddot{z}}_{u}-F_{k}-F_{c}+k_{t}(z_{u}-z_{r})=0 \end{array}\right.$$

where $m_{s}$ is the sprung mass, $m_{u}$ is the unsprung mass, $k_{s}$ is the equivalent stiffness of the spring, $k_{t}$ is the equivalent stiffness of the tire, $c_{s}$ is the equivalent damping coefficient of the suspension, $z_{s},z_{u}$ is the displacement of the sprung mass, and $z_{r}$ is the road elevation.

Substituting Equations (3) and (4) into Equation (5), the dynamic equations of the nonlinear suspension system can be summarized as follows:

(6) $$\left\{\begin{array}{c} \begin{array}{l} m_{s}{\ddot{z}}_{s}+k_{s}^{l}(z_{s}-z_{u})+k_{s}^{n}{\left(z_{s}-z_{u}\right)}^{3}\\ +c_{s}^{l}\left({\dot{z}}_{s}-{\dot{z}}_{u}\right)+c_{s}^{a}\left|{\dot{z}}_{s}-{\dot{z}}_{u}\right|+c_{s}^{n}\sqrt{\left|{\dot{z}}_{s}-{\dot{z}}_{u}\right|}\mathrm{sgn}\left({\dot{z}}_{s}-{\dot{z}}_{u}\right)=0 \end{array}\\ \begin{array}{l} m_{u}{\ddot{z}}_{u}-k_{s}^{l}(z_{s}-z_{u})-k_{s}^{n}{\left(z_{s}-z_{u}\right)}^{3}-c_{s}^{l}\left({\dot{z}}_{s}-{\dot{z}}_{u}\right)\\ +c_{s}^{a}\left|{\dot{z}}_{s}-{\dot{z}}_{u}\right|+c_{s}^{n}\sqrt{\left|{\dot{z}}_{s}-{\dot{z}}_{u}\right|}\mathrm{sgn}\left({\dot{z}}_{s}-{\dot{z}}_{u}\right)+k_{t}(z_{u}-z_{r})=0 \end{array} \end{array}\right.$$

The Table 2 below presents the key parameters of the nonlinear suspension system.

## 3. Road Roughness Identification Based on NARX Neural Network

### 3.1. NARX Network Construction

The vibration acceleration signal of a vehicle is a typical sequential signal. Traditional classification methods often involve manual feature extraction from these sequential signals, utilizing various features as input data. While an increased number of features can enhance the classification accuracy, it also significantly elevates the computational complexity and may lead to the curse of dimensionality. The time series acceleration signals inherently contain different features.

**Figure 2:**
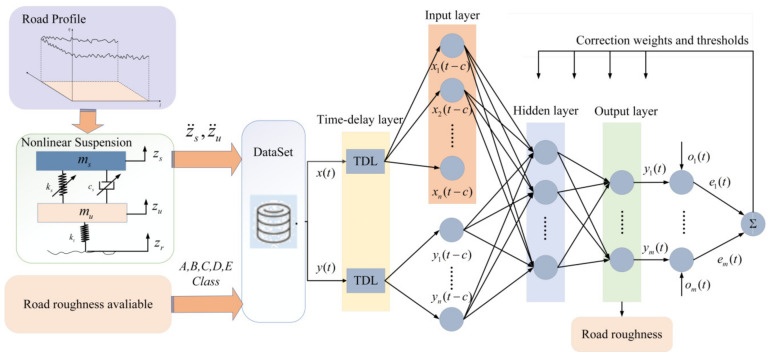
NARX network with an open-loop structure.

However, traditional static neural networks, such as BP, cannot effectively handle sequential signals with interdependent relationships. In contrast, NARX neural networks are particularly adept at processing these types of data. Therefore, this paper selects the NARX neural network as the preferred model for this analysis. The training network and the main structure are illustrated in Figure 2.

The TDL represents the delay processing, which applies delays to both the inputs x(t) and outputs y(t), feeding them back into the input layer. The input layer can set weights and thresholds for the inputs and relay this information to the hidden layer, with the number of neurons in the input layer denoted as *n*. The output layer computes the output values y(t) based on feedback from the hidden layer, compares them with the desired output values o(t) to calculate the error, and feeds this information back to both the input and hidden layers to adjust and update the weights and thresholds; the number of neurons in the output layer is also *n*.

**Figure 3:**
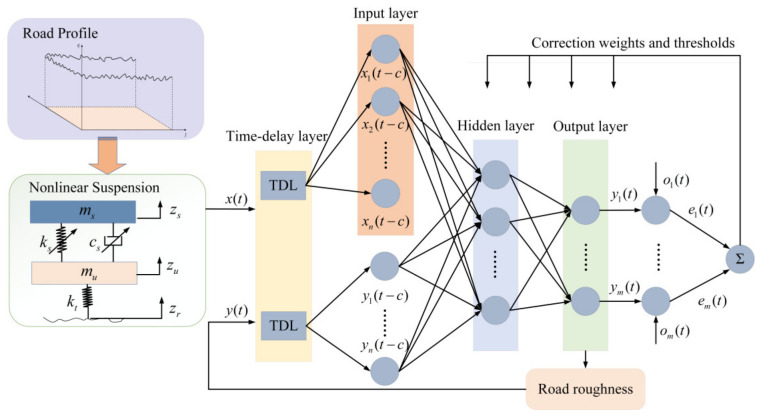
NARX network with a closed-loop structure.

The NARX neural network has two structures [22]: one is the closed-loop structure, where the actual output of the neural network is fed back to the input, as shown in Figure 3; the other is the open-loop structure, where the desired output (rather than the actual output of the neural network) is fed back to the input, significantly improving the network’s prediction performance, as shown in Figure 3. When output data are available, the neural network is trained in the open-loop form and then switched to the closed-loop form, providing only external inputs for multi-step prediction.

### 3.2. Design of Road Roughness Identification Based on NARX

This paper utilizes the dynamic response values of a nonlinear suspension system as training targets. The number of neurons in the time delay layer, input layer, and output layer of the NARX neural network is determined based on the specific inputs and outputs of the problem at hand. Generally, the output is delayed only once. To reduce the computational complexity for practical implementation, the number of hidden layers mmm is set to one. Additionally, the number of neurons in the hidden layer can be calculated using the following Equation (7) [23]:

(7) $$l=\sqrt{m+n}+a$$

where *a* is a constant factor in the range of numbers 1–10.

The transfer function f of the hidden layer is set as logsig, and the transfer function g of the output layer is set as purelin:

(8) $$\left\{\begin{array}{l} f(x)=\frac{1}{1+e^{-x}}\\ g(x)=x \end{array}\right.$$

The weights and thresholds of the neural network are adjusted by the training algorithm, where the default training algorithm is trainlm, which has a fast training speed and high identification accuracy [23].

The response quantities correlated with road roughness in a quarter vehicle model are the sprung mass acceleration ${\ddot{z}}_{s}$, unsprung mass acceleration ${\ddot{z}}_{u}$, suspension dynamic deflection $z_{s}-z_{u}$, and wheel dynamic load $k_{t}(z_{u}-z_{r})$. As the wheel dynamic load is challenging to obtain directly from the vehicle and the dynamic deflection primarily reflects the deformation of the suspension which does not directly indicate changes in the road conditions, the sprung and unsprung mass acceleration are selected as the training inputs. At the same time, road roughness serves as the output.

Considering that the typical speed on normal urban roads ranges from 25 to 50 km/h, this paper selects a speed of 30 km/h for the dataset creation, and 5000 sets of vehicle response data are obtained under randomly generated road conditions ranging from Class A to E. For the open-loop NARX network training process, the model is configured with two NARX layers. The input delay is 1:2, and the feedback delay is 1:2. The hidden layer consists of 15 neurons, with one fully connected layer. The training goal is set to 1 × 10^−8^, and the minimum gradient is 1 × 10^−5^. The training process uses the ADAM optimizer and runs for 250 epochs. The training function is trainlm. Finally, the neural network is trained using 70% of the dataset as the training set, 15% as the validation set, and 15% as the test set.

## 4. Road Roughness Identification Based on NARX-PASCKF

### 4.1. The SCKF Estimator for Road Roughness Identification

In practical engineering applications, a series of Kalman filtering algorithms, including KF, EKF, UKF, and particle filtering, are widely used for state and parameter estimation in dynamic systems. KF is particularly suited to linear systems but lacks applicability for nonlinear systems. In contrast, EKF addresses this limitation by linearizing the system through the computation of the Jacobian matrix, thereby extending its applicability to nonlinear systems. However, when the system is highly nonlinear, linearization can lead to significant truncation errors, and the computation of the Jacobian matrices for the state and observation equations can be challenging, severely affecting the estimation accuracy [24].

UKF employs the unscented transformation to handle the nonlinear propagation of the means and covariances, approximating the probability density distribution of the nonlinear functions. It offers higher accuracy than EKF; however, in high-dimensional and strongly nonlinear systems, the negative weights of the central sampling points can lead to significant biases, posing challenges to ensuring the stability of the estimation results [25].

CKF approximates the Gaussian-weighted integrals in strongly nonlinear systems using a third-order spherical simplex rule. It accurately models the system dynamics and measurement noise and provides advantages such as lower computational complexity, faster convergence, and stronger robustness [26].

SCKF is a novel filtering method based on CKF, founded on the principle of square root filtering. Compared to CKF, SCKF reduces the computational complexity and enhances the filtering efficiency and convergence speed [27].

Consider the following nonlinear system:

(9) $$\left\{\begin{array}{l} \dot{x}(t)=f[x(t)]+W(t)\\ z(t)=h[x(t)]+V(t) \end{array}\right.$$

where z(t) indicates the observation variables, and $V(t)={\left[v_{1}(t),v_{2}(t)\right]}^{T}$ represents the measurement noise, also $v_{i}(t)$ follows a zero-mean Gaussian distribution v(t)~(0,R), x(t) represents the state variables, $W(t)={\left[\theta_{1}(t),\theta_{2}(t),\theta_{3}(t),\theta_{4}(t),2\pi n_{0}\sqrt{G_{q}(n_{0})v}w(t)\right]}^{T}$ denotes the process noise, and θ(t) follows a zero-mean Gaussian distribution $\theta_{i}(t)\sim (0,Q)$. In this paper, the state variables are selected as $x(t)={\left[z_{s},{\dot{z}}_{s},z_{u},{\dot{z}}_{u},z_{r}\right]}^{T}$, and the observation variables are chosen as $z(t)={\left[{\ddot{z}}_{s},{\ddot{z}}_{u}\right]}^{T}$.

The above continuous equations can be discretized as Equation (10).

(10) $$\left\{\begin{array}{l} {\dot{x}}_{k}=f(x_{k-1})+W_{k-1}\\ z_{k}=h(x_{k})+V_{k} \end{array}\right.$$

From Equation (10), it follows that the system can be described by the following state update Equation (11) and measurement Equation (12).

(11) $$f(x_{k-1})=\left(\begin{array}{c} x_{2,k-1}\\ -\frac{F_{k,k-1}}{m_{s}}-\frac{F_{c,k-1}}{m_{s}}\\ x_{4,k-1}\\ \frac{F_{k,k-1}}{m_{u}}+\frac{F_{c,k-1}}{m_{u}}-\frac{k_{t}}{m_{u}}(x_{3,k-1}-x_{5,k-1})\\ -2\pi n_{c}vx_{5,k-1} \end{array}\right)$$

(12) $$h(x_{k})=\left(\begin{array}{c} -\frac{F_{k,k}}{m_{s}}-\frac{F_{c,k}}{m_{s}}\\ \frac{F_{k,k-1}}{m_{u}}+\frac{F_{c,k-1}}{m_{u}}-\frac{k_{t}}{m_{u}}(x_{3,k}-x_{5,k}) \end{array}\right)$$

where $F_{k,k-1}$ and $F_{c,k-1}$ are expressed by the following equations:

(13) $$\left\{\begin{array}{ll} F_{k,k-1} & =k_{s}^{l}(z_{s,k-1}-z_{u,k-1})+k_{s}^{n}{\left(z_{s,k-1}-z_{u,k-1}\right)}^{3}\\ & =k_{s}^{l}(x_{1,k-1}-z_{3,k-1})+k_{s}^{n}{\left(z_{1,k-1}-z_{3,k-1}\right)}^{3}\\ F_{c,k-1} & =c_{s}^{l}\left({\dot{z}}_{s,k-1}-{\dot{z}}_{u,k-1}\right)+c_{s}^{a}\left|{\dot{z}}_{s,k-1}-{\dot{z}}_{u,k-1}\right|\\ & +c_{s}^{n}\sqrt{\left|{\dot{z}}_{s,k-1}-{\dot{z}}_{u,k-1}\right|}\mathrm{sgn}\left({\dot{z}}_{s,k-1}-{\dot{z}}_{u,k-1}\right)\\ & =c_{s}^{l}\left(x_{2,k-1}-x_{4,k-1}\right)+c_{s}^{a}\left|x_{2,k-1}-x_{4,k-1}\right|\\ & +c_{s}^{n}\sqrt{\left|x_{2,k-1}-{\dot{z}}_{4,k-1}\right|}\mathrm{sgn}\left(x_{2,k-1}-{\dot{z}}_{4,k-1}\right) \end{array}\right.$$

In the discrete nonlinear system, the process noise $W_{k-1}$ and the measurement noise $V_{k}$ are uncorrelated zero-mean Gaussian white noise, with their uncorrelated covariances described as:

(14) $$\left\{\begin{array}{l} E\left(W_{k-1},W_{j-1}^{T}\right)=Q_{k-1}\\ E\left(V_{k},v_{j}^{T}\right)=R_{k}\delta_{kj} \end{array}\right.$$

where $Q_{k-1}$ is the non-negative definite matrix of process noise, $R_{k}$ is the positive definite measurement covariance matrix, and $\delta_{kj}$ is the Kronecker delta function.

Thus, the standard SCKF algorithm can be summarized as follows:

(1) Initialization

Assume the initial state at time $t_{0}$ is ${\hat{x}}_{0\left|0\right.}$ and the corresponding square root factor of the error covariance matrix is $S_{0\left|0\right.}$, where the expression for $S_{0\left|0\right.}$ is:

(15) $$S_{0\left|0\right.}={\left[Chol(P_{0\left|0\right.})\right]}^{T}$$

where Chol is the Cholesky decomposition, and $P_{0\left|0\right.}$ is the error covariance matrix.

(2) Time update

Calculate the sigma points and propagate them based on the state transition equation:

(16) $$\left\{\begin{array}{l} x_{k-1\left|k-1\right.}^{i}={\hat{x}}_{k-1\left|k-1\right.}+S_{k-1\left|k-1\right.}\xi_{i}\begin{array}{cc} & i=1,2,\cdots \end{array},2n\\ x_{k-1\left|k-1\right.}^{i*}=f(x_{k-1\left|k-1\right.}^{i}) \end{array}\right.$$

where $\xi_{i}$ is the i-th column of the weight matrix $\left[\sqrt{n}I_{n},-\sqrt{n}I_{n}\right]$ for the sigma points, $I_{n}$ is the identity matrix of n×n, and n is the dimension of the state variables.

Calculate the predicted state values and the square root factor of the error covariance matrix:

(17) $$\left\{\begin{array}{l} {\hat{x}}_{k\left|k-1\right.}=\frac{1}{2n}\sum_{i=1}^{2n}x_{k\left|k-1\right.}^{i*}\\ S_{k\left|k-1\right.}=Tria(\left[X_{k\left|k-1\right.}^{i*},Chol(Q_{k-1})\right]) \end{array}\right.$$

The weighted central matrix $X_{k\left|k-1\right.}^{i*}$ is defined as:

(18) $$X_{k\left|k-1\right.}^{i*}=\frac{1}{\sqrt{2n}}\left[x_{k\left|k-1\right.}^{1*}-{\hat{x}}_{k\left|k-1\right.},x_{k\left|k-1\right.}^{2*}-{\hat{x}}_{k\left|k-1\right.},\cdots ,x_{k\left|k-1\right.}^{2n*}-{\hat{x}}_{k\left|k-1\right.}\right]$$

where the operation of Tria(⋅) is defined as follows: let R be the upper triangular matrix of the QR decomposition of matrix $A^{T}$, then the QR decomposition algorithm of matrix A can be expressed as $S=Tria(\cdot )=R^{T}$, and S is the lower triangular matrix.

(3) Measurement update

Update the sigma points and propagate them based on the measurement equation:

(19) $$\left\{\begin{array}{l} x_{k\left|k-1\right.}^{i}={\hat{x}}_{k\left|k-1\right.}+S_{k\left|k-1\right.}\xi_{i}\\ z_{k\left|k-1\right.}^{i}=h(x_{k\left|k-1\right.}^{i}) \end{array}\right.$$

Calculate the predicted measurement values and the square root factor of the innovation covariance matrix:

(20) $$\left\{\begin{array}{l} {\hat{z}}_{k\left|k-1\right.}=\frac{1}{2n}\sum_{i=1}^{2n}z_{k\left|k-1\right.}^{i}\\ S_{k\left|k-1\right.}^{zz}=Tria(\left[Z_{k\left|k-1\right.},Chol(R_{k})\right]) \end{array}\right.$$

Similarly, the weighted central matrix $Z_{k|k-1}$ is defined as:

(21) $$Z_{k|k-1}=\frac{1}{\sqrt{2n}}\left[z_{k|k-1}^{1}-{\hat{z}}_{k|k-1},z_{k|k-1}^{2}-{\hat{z}}_{k|k-1},\cdots ,z_{k|k-1}^{2n}-{\hat{z}}_{k|k-1}\right]$$

Calculate the measurement covariance matrix and the cross-covariance matrix:

(22) $$\left\{\begin{array}{l} P_{k\left|k-1\right.}^{zz}=S_{k\left|k-1\right.}^{zz}{\left(S_{k\left|k-1\right.}^{zz}\right)}^{T}\\ P_{k\left|k-1\right.}^{xz}=X_{k\left|k-1\right.}Z_{k\left|k-1\right.}^{T} \end{array}\right.$$

The weighted central matrix is defined as follows:

(23) $$X_{k|k-1}=\frac{1}{\sqrt{2n}}\left[x_{k|k-1}^{1}-{\hat{x}}_{k|k-1},x_{k|k-1}^{2}-{\hat{x}}_{k\left|k-1\right.},\cdots ,x_{k\left|k-1\right.}^{2n}-{\hat{x}}_{k\left|k-1\right.}\right]$$

(4) Calculation of the Kalman gain matrix

After the above steps, the final expression for the Kalman gain matrix is the following:

(24) $$K_{k}=P_{k\left|k-1\right.}^{xz}{\left(P_{k\left|k-1\right.}^{zz}\right)}^{-1}$$

Finally, the updated state variables and the error covariance matrix are as follows:

(25) $$\left\{\begin{array}{l} {\hat{x}}_{k\left|k\right.}={\hat{x}}_{k\left|k-1\right.}+K_{k}(z_{k}-{\hat{z}}_{k\left|k-1\right.})\\ S_{k\left|k\right.}=Tria\left(\left[X_{k\left|k\right.}-K_{k}Z_{k\left|k-1\right.},K_{k}Chol(R_{k})\right]\right) \end{array}\right.$$

**Figure 4:**
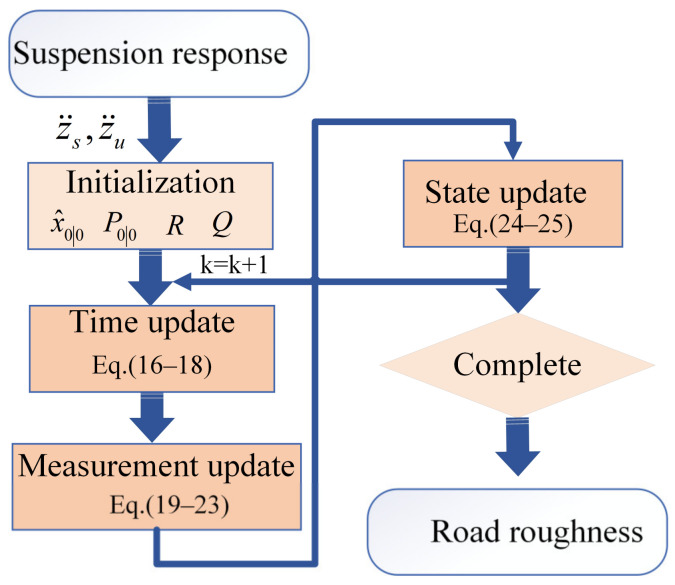
Estimation of road roughness based on SCKF.

The SCKF road roughness identification method, based on the nonlinear suspension model, requires that the vehicle states satisfy the dynamic equations to achieve high estimation accuracy. The process of road roughness identification is illustrated in Figure 4. Observations obtained from sensors, including the sprung and unsprung mass acceleration, are input into the SCKF estimator. Finally, the output is the estimated road roughness.

### 4.2. Design of NARX-PASCKF Algorithm for Road Roughness Identification

**Figure 5:**
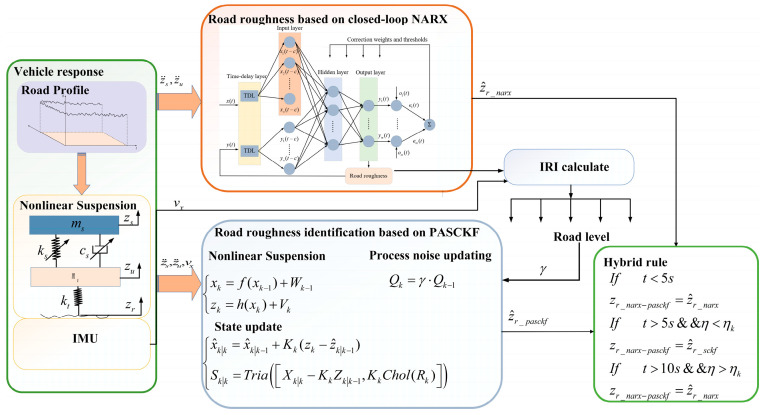
Estimation of road roughness based on NARX-PASCKF.

Although the road roughness identification based on dynamic models achieves good estimation accuracy, this approach is susceptible to varying operating conditions. For instance, during continuous road surface changes, the continuous variation of the process covariance can lead to a decline in the estimation accuracy of the SCKF model. Additionally, the SCKF state estimator based on dynamic models is affected by the initial states and constant noise parameters, resulting in longer convergence times. To address these issues, this paper proposes a process noise adaptive square root cubature Kalman filter (PASCKF) and designs a fusion estimation strategy by combining it with the NARX network to estimate the road classification. The basic framework is illustrated in Figure 5.

The suspension response is obtained from sensors, and the vehicle speed is obtained by integrating the x-axis signal of the IMU. In Kalman filtering, the noise covariance matrix *Q* represents the covariance of the process noise, describing the level of uncertainty or model error within the system model. The size of this matrix reflects the intensity of the process noise introduced for each state variable. A larger matrix indicates a more significant uncertainty in the system’s state changes. In comparison, a smaller matrix suggests that the state changes are more specific and the model is more reliable.

As the vehicle travels on different grades of road surfaces, the noise generated can vary significantly. Therefore, it is essential to consider the variations in the noise covariance matrix Q caused by other road surfaces, introducing adjustments to better account for these effects.

(26) $$Q_{k}=\gamma Q_{k-1}$$

where, $Q_{k}$ represents the process covariance matrix updated for the next time step, and γ denotes the updated covariance factor matrix. This study conducts simulation experiments at a vehicle speed of 30 km/h over road surfaces graded A to E. Using the covariance formula, the relationship can be expressed as follows:

(27) $$Cov(X)=\frac{1}{N}\sum_{i=1}^{N}\left(X_{i}-\mu \right){\left(X_{i}-\mu \right)}^{T}$$

where, X represents the vehicle response values, μ is the mean of the vehicle response values, and N is the number of sample data points. After calculating the covariance, the vehicle response covariance values for each grade of the road surface are shown in Table 3.

Considering that the measurement covariance matrix is primarily related to the actual sensor accuracy, this study employs automotive-grade sensors with an error magnitude of approximately 0.1%. Therefore, the error covariance *R*_k_ is set to 0.001^2^. The above adjustments represent the correction of the process noise and measurement noise for the PASCKF estimator. The following section proposes a strategy based on the fusion of NARX and PASCKF.

(1) To address the issue of slow convergence of the PASCKF at the initial moment, during the first 5s, the NARX neural network is used for estimation. Simultaneously, it outputs γ to the PASCKF estimator to correct the process noise $Q_{k}$ for the next time step, thereby improving the estimation accuracy and convergence speed of the PASCKF estimator.

(2) To address the accuracy issue of the PASCKF estimator, a variable η is introduced, defined by the Equation (28).

(28) $$\eta =\frac{\left|{\ddot{z}}_{s\_cal}-{\ddot{z}}_{s\_sen}\right|}{\left|{\ddot{z}}_{s\_sen}\right|}$$

where, ${\ddot{z}}_{s\_cal}$ represents the acceleration value obtained by differentiating the sprung mass velocity estimated by the PASCKF. ${\ddot{z}}_{s\_sen}$ is the sprung mass acceleration value obtained from the sensor. Set a threshold $\eta_{k}$ = 0.1. If $\eta <\eta_{k}$, it indicates that the values calculated by the PASCKF are accurate. In this case, the NARX-PASCKF will directly output the values from the PASCKF. Additionally, if t > 5s && $\eta >\eta_{k}$, considering the possibility of estimation inaccuracies in the volume Kalman filter, the NARX will be used for the output.

(3) Considering the issue where the PASCKF fails to converge and correctly identify the road surface, if it does not converge within 10 s and the NARX is able to identify the road surface stably, the NARX-PASCKF will then directly output the values from the NARX.

## 5. Simulation Experiments

### 5.1. Road Roughness Identification Results Based on the NARX Algorithm

The NARX road roughness estimator provides process noise correction factors and the estimated values for the first 5 s to the NARX-PASCKF, which is critical for improving the estimation accuracy and convergence speed of the PASCKF. Therefore, this section will validate the estimation accuracy of the NARX model. The test was conducted by selecting a vehicle speed of 36 km/h on C grade road. The testing durations were set to 10 s for each grade. The test datasets were input into both the NARX and BP neural networks for comparative validation.

Additionally, the root mean square error (RMSE), mean absolute error (MAE), and coefficient of determination (R-square) were compared with Equations (29)–(31).

(29) $$RMSE=\sqrt{\frac{\sum_{i=1}^{N}{\left(q_{i}-o_{i}\right)}^{2}}{N}}$$

(30) $$MAE=\frac{1}{N}\sum_{i=1}^{N}\left|q_{i}-o_{i}\right|$$

(31) $$R^{2}=1-\frac{\sum_{i=1}^{n}{\left(o_{i}-{\hat{q}}_{i}\right)}^{2}}{\sum_{i=1}^{n}{\left(o_{i}-\bar{o}\right)}^{2}}$$

where, $q_{i}$ represents the estimated road roughness, $o_{i}$ is the actual road roughness, $\bar{q}$ and $\bar{o}$ represent the identified road roughness mean and the actual road roughness mean, *N* is the total number of sampling points.

**Figure 6:**
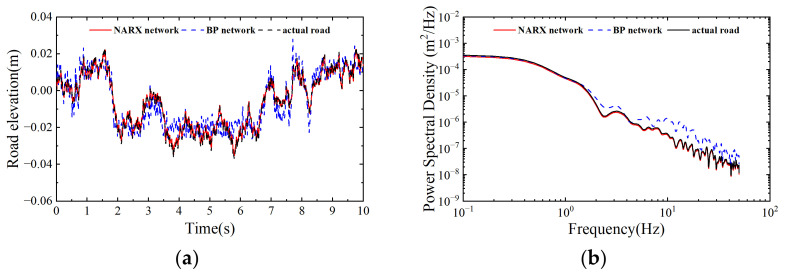
Neural network recognition results. (**a**) Road elevation results. (**b**) Power spectral density comparison.

The experimental results are shown in Figure 6 and Table 4.

Figure 6a,b show the road roughness estimated by the NARX network and the BP network. It can be seen that the BP neural network can identify the basic profile of the road roughness, but due to its inability to capture the temporal relationships in acceleration signals, its estimation accuracy is significantly lower than that of the NARX neural network.

Table 4 compares the RMSE, MAE, and the coefficient R-square between the NARX network and the BP network. The NARX network achieves smaller root mean square values and mean absolute error values compared to the BP network, with the estimation accuracy improving by 46.42% and 43.39%, respectively. Additionally, the determination coefficient R-square has also increased by 26.61%. The above results demonstrate that the NARX neural network can estimate road roughness more accurately compared to the BP neural network, providing a solid foundation for improving the subsequent NARX-PASCKF algorithm.

### 5.2. Single Random Road Surface Experiment

To validate the identification accuracy of the proposed NARX-PASCKF hybrid estimation algorithm, simulation tests were conducted on a single random road surface and on connecting road surfaces A–B–D–C. The test results were compared with the actual road surfaces and the results from the single SCKF estimator.

**Figure 7:**
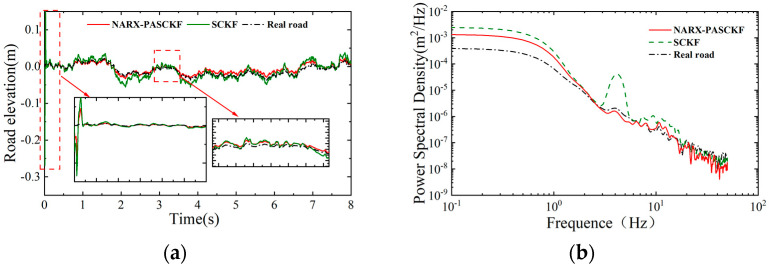
C-class road recognition results. (**a**) Road elevation results. (**b**) Power spectral density comparison.

Using a 36 km/h speed on a C-grade road surface, random road roughness signals were generated using Equation (1), and simulation tests were conducted under steady-state conditions. The test results were compared with the actual road surface and the results from the standalone SCKF estimator. The final experimental results are shown in Figure 7 and Table 5.

Figure 7a shows the road roughness results of the SCKF and the NARX-PASCKF estimator and their corresponding PSD, which are shown in Figure 7b. The results show that the SCKF estimator can estimate the general profile and the trend of the strengthened road roughness. However, there is a discrepancy between the estimated and measured results at the initial moment. In the frequency plot, the PSD estimation results from the SCKF exhibit discrepancies compared to the actual results, particularly in the frequency range of 1.5–11 Hz (0.15–1.1 m^−1^), where the PSD values of the road roughness estimated by the SCKF are significantly higher than the actual road roughness PSD values. These estimation errors may be due to the neglect of the appropriate noise covariance, and the issue can be addressed by the proposed NARX-PASCKF estimator.

It can be seen in Figure 7a,b that the road roughness estimated by the NARX-PASCKF estimator is closer to the actual road roughness. In comparison with the SCFK estimator, the NARX-PASCKF estimator exhibits a smaller convergence amplitude, leading to more stable and accurate estimations. In the frequency plot, the estimated PSD value of the NARX-PASCKF estimator is more consistent with the actual PSD value in the spatial frequency range of 1.5–11 Hz (0.15–1.1 m^−1^).

Furthermore, Table 5 shows the RMSE, MAE, and the coefficient R-square between the SCKF estimator and the NARX-PASCKF estimator, which provide a quantitative reference for evaluating the validity of the estimation. The results show that the NARX-PASCKF algorithm achieves lower root mean square values and mean absolute error values compared to the standalone SCKF estimator, with estimation accuracy improvements of 30.64% and 24.7%, respectively. In terms of the coefficient R-square, the NARX-SCKF algorithm also outperforms the standalone SCKF algorithm, showing an accuracy improvement of 6.3%. The results show that the hybrid model of the NARX-PASCKF can effectively improve the road roughness estimation accuracy compared with the single SCKF.

### 5.3. Joint Road Surface Experiment

**Figure 8:**
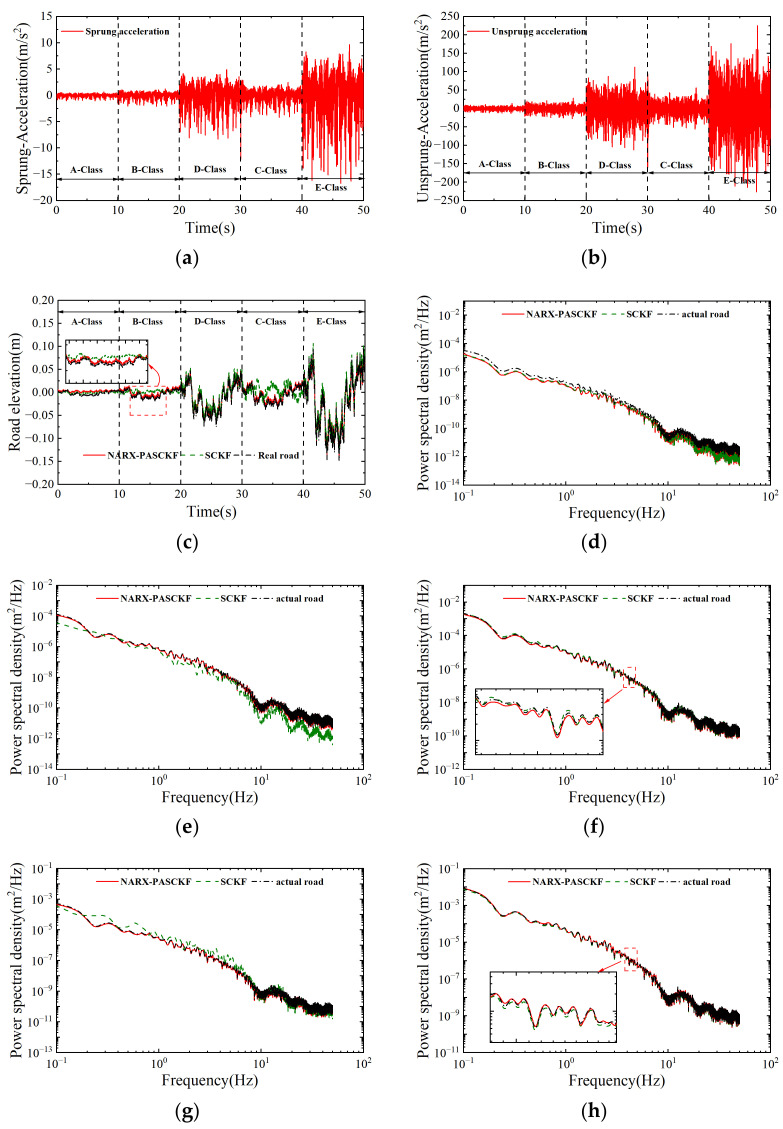
Joint road recognition results. (**a**) Data on sprung acceleration. (**b**) Data of unsprung acceleration. (**c**) Road elevation results. (**d**) Power spectral density results on A class. (**e**) Power spectral density results on B class. (**f**) Power spectral density results on D class. (**g**) power spectral density results on C class. (**h**) Power spectral density results on E class.

To verify the adaptability of the NARX-SCKF hybrid estimation algorithm, variable-grade joint road surfaces (A–B–D–C–E) were selected for the simulation testing. The simulation time for each grade of road surface was set to 10 s, with a vehicle speed of 36 km/h, resulting in a total duration of 50 s. The results are shown in Figure 8 and Table 6.

Figure 8a,b show the sprung and unsprung mass acceleration responses under different road surfaces. The acceleration response values have a specific amplitude range for different road grades. Figure 8c shows the road roughness results of the SCKF and the NARX-PASCKF estimator and their corresponding PSD, which are shown in Figure 8b–h. Similar to the single road surface experiment, the result of the joint road experiment shows that the SCKF estimator can estimate the general profile and the trend of the strengthened road roughness. However, the detailed estimation of road roughness still requires further improvement. In the frequency plot, the SCKF estimator performs reasonably well on D-grade and E-grade roads. However, when the road surface changes to B grade, its PSD values remain below the actual road PSD values in the frequency range of 10^0^–10^2^ Hz (0.1–10 m^−1^). Conversely, when the road changes to C grade, its PSD values are consistently higher than the actual road PSD values in the frequency range of 10^0^–10^1^ Hz (0.1–1 m^−1^).

Figure 8c–h show that the road roughness estimated by the NARX-PASCKF estimator is closer to the actual road roughness than the SCFK estimator. Due to the introduction of process noise factors and the hybrid strategy, the results in Figure 8e,g indicate that the estimated PSD values of road roughness in the frequency ranges of 10^0^–10^2^ Hz (0.1–10 m^−1^) and 10^0^–10^1^ Hz (0.1–1 m^−1^) have been corrected.

Table 6 compares the RMSE, MAE, and the coefficient R-square between the SCKF estimator and the NARX-PASCKF estimator. The NARX-PASCKF hybrid algorithm achieves smaller root mean square values and mean absolute error values compared to the single SCKF estimator, with the estimation accuracy improving by 31.7% and 41.5%, respectively. Additionally, the determination coefficient R-square has also increased by 9.04%.

The above results indicate that the NARX-PASCKF estimator can effectively identify road roughness under various road conditions, and its identification performance is superior to that of the single SCKF estimator. Therefore, the NARX-PASCKF algorithm proposed in this paper demonstrates good adaptability.

## 6. Actual Car Experiments

### 6.1. Experimental Platform Setup

**Figure 9:**
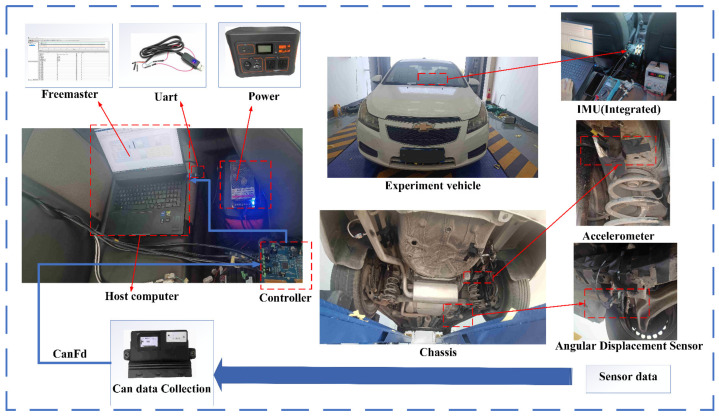
Communication structure diagram.

This paper conducted actual vehicle experiments to verify the effectiveness of the NARX-PASCKF estimation of road roughness. On the right side of Figure 9 is the test vehicle (Chevrolet Cruze) and the installation positions of the sensors. The IMU is mounted at the vehicle’s center of mass, which is a six-axis sensor with a frequency of 100 Hz. The sprung mass accelerometer is installed on the chassis, and the angular displacement sensor has one end on the chassis and the other on the wheel. This setup can calculate the unsprung mass acceleration signal. Finally, the road information for the test was collected by a laser rangefinder (provided by Arctic Technology, Guangdong, China), with a frequency range of 1–20 Hz.

The communication process of the signals is shown on the right side of Figure 9. The sensor signals are transmitted via CAN communication from the sensors to the suspension controller, and finally, through serial communication, they are transmitted in real time to the host computer. The data signals are then observed and read using Freemaster (NXP).

### 6.2. Real Vehicle Validation

**Figure 10:**
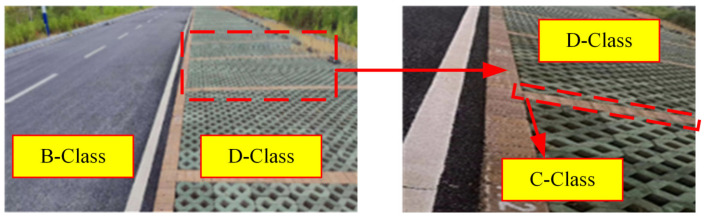
Test road.

The experiment uses a section of smooth asphalt road and a bumpy road for validation. The roughness level of the asphalt road is near grade B, while the rough road is around grade D. The road images are shown in Figure 10. The vehicle drives over both types of roads at a speed of 20 km/h, collecting data for 4 s on each road, with a sampling time of 0.01 s, resulting in 800 data samples.

**Figure 11:**
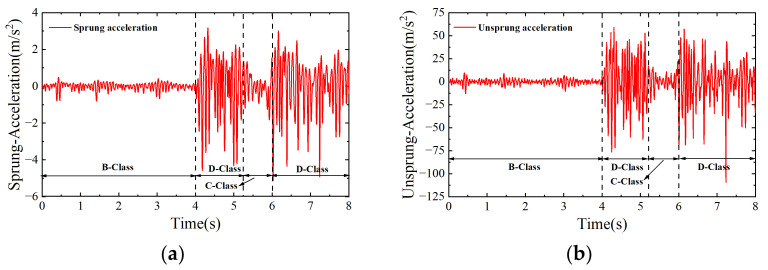

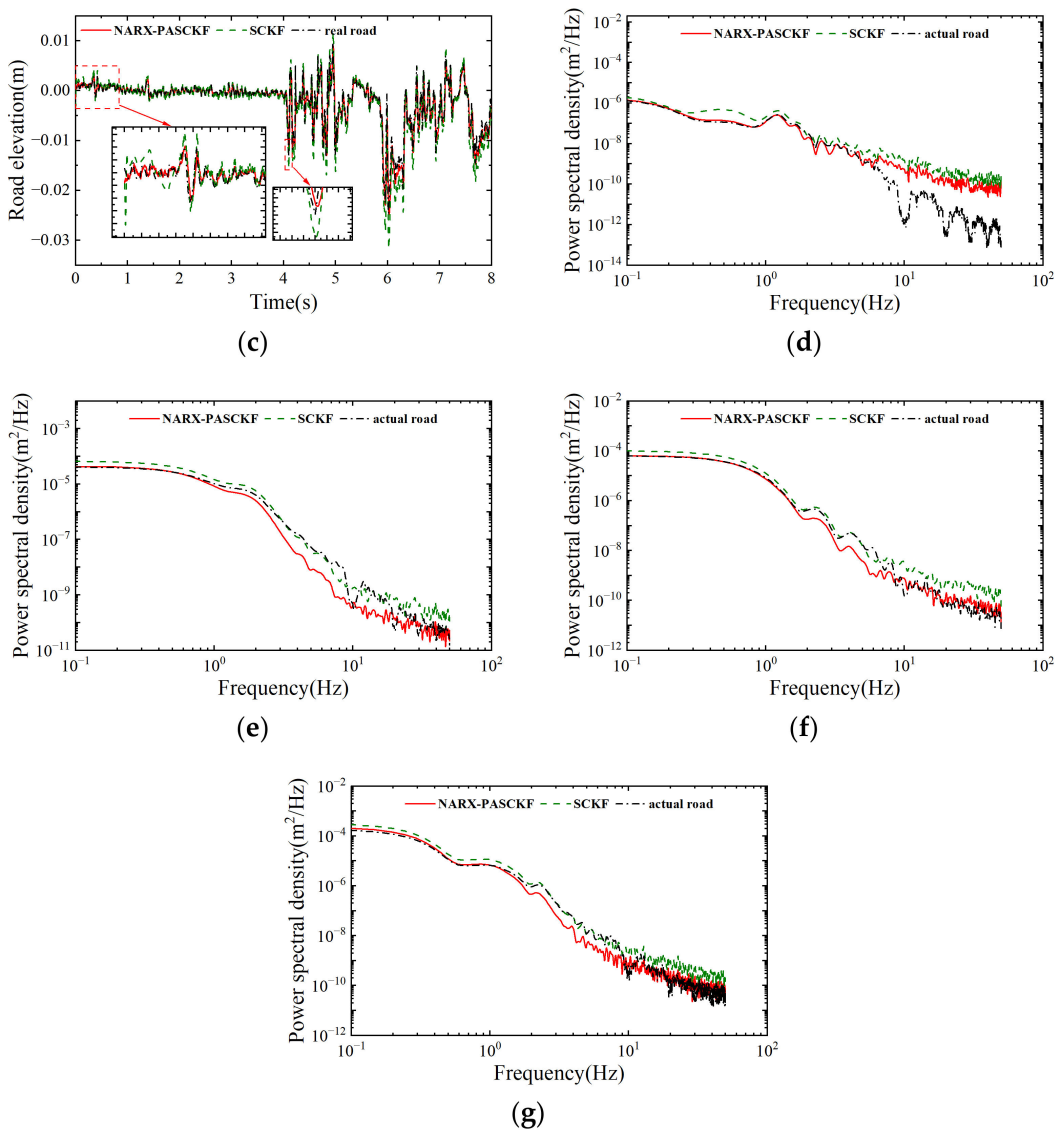
Actual joint road recognition results. (**a**) Data on sprung acceleration. (**b**) Data of unsprung acceleration. (**c**) Road elevation results. (**d**) Power spectral density results on B class. (**e**) Power spetral density results on D class 1. (**f**) Power spectral density results on C class. (**g**) Power spectral density results on D class 2.

After collecting real-time road–vehicle responses, the actual road conditions and the road results identified from the SCKF and NARX-PASCKF are compared. The vehicle response data and road recognition results are shown in Figure 11.

Figure 11a,b show the spring upper acceleration response values and spring lower acceleration response values under different road surfaces. It is evident that three distinct road grades are present: B grade in the first segment, D grade in the second segment, and gaps consisting of C-grade brick surfaces.

Figure 11c shows the road roughness results of the SCKF and the NARX-PASCKF estimator and their corresponding PSD, which are shown in Figure 11d–g. Similar to the simulation, the result shows that the SCKF estimator can estimate the general profile and the trend of the strengthened road roughness. However, further corrections are still needed in the detailed estimation of the roughness variations. In the frequency plot, the PSD estimation results from the SCKF exhibit discrepancies compared to the actual results, particularly in the frequency range of 10^1^–10^2^ Hz (1–10 m^−1^), where the PSD values of the road roughness estimated by the SCKF are significantly higher than the actual road roughness PSD values.

Figure 11c–g show that the road roughness estimated by the NARX-PASCKF estimator is closer to the actual road roughness than the SCFK estimator. Due to the introduction of process noise factors and the hybrid strategy, the results in Figure 11d–g indicate that the estimated PSD values of road roughness in the frequency ranges of 10^1^–10^2^ Hz (1–10 m^−1^) have been corrected.

Table 7 compares the RMSE, MAE, and the coefficient R-square between the SCKF estimator and the NARX-PASCKF estimator. The RMSE of the NARX-PASCKF algorithm is reduced by 20.75%, the RMSE of the NARX-PASCKF algorithm is reduced by 26.56% and the coefficient R-square of the NARX-PASCKF algorithm is improved by 2.4%.

The real-vehicle experimental data indicate that the proposed NARX-PASCKF hybrid algorithm is an effective method for identifying road roughness. In particular, the NARX-PASCKF algorithm improves the covariance matrix of the PASCKF by integrating the estimates from the NARX neural network, thereby enhancing the estimation accuracy compared to the SCKF. However, as shown in Figure 11e–g, there are still certain discrepancies between the NARX-PASCKF estimation algorithm and the actual road conditions in the frequency range of 1–10 Hz (0.1–1 m^−1^). These discrepancies may be attributed to either the vehicle model or the overfitting issue of the NARX when predicting unknown road surfaces. In this paper, the nonlinearity of the vehicle model parameters is approximated, and the training of the NARX neural network is conducted using simulation data. This approach may lead to overfitting issues when faced with unknown road surfaces. Therefore, further research is needed to address the challenges of accurate vehicle modeling and to mitigate the overfitting problem in neural network predictions.

## 7. Conclusions

To address the challenge of accurately identifying road roughness due to the strong nonlinearity of the suspension system, this paper proposes an NARX-PASCKF hybrid identification method. The proposed method is based on both the NARX neural network and the SCKF. First, nonlinear suspension road roughness estimation models are constructed using the NARX neural network and SCKF estimators. Next, to account for the impact of changes in the process noise covariance during road surface variations, a process noise factor correction is introduced, resulting in the process noise adaptive square root cubature Kalman filter (PASCKF). Finally, considering the convergence and estimation accuracy issues of the PASCKF, the method proposes to use the road roughness estimated by the NARX to convert the road type into a process noise factor for real-time correction, along with a corresponding fusion strategy to ensure the accuracy of the estimation.

The effectiveness and adaptability of the process are validated through simulation and real-vehicle experiments. The actual vehicle experiments demonstrate that under the condition of the joint road, the root mean square error (RMSE) of the NARX-PASCKF estimator is reduced by 20.75%, the mean absolute error (MAE) is decreased by 26.56%, and the determination coefficient R-square is improved by 5.1% compared to the single SCKF estimator. These results indicate that the proposed NARX-PASCKF algorithm can accurately estimate road roughness.

In practice, compared to direct contact measurements and measurements, the estimation method proposed in this paper is more economical and versatile. The NARX-PASCKF estimator based on the nonlinear suspension model proposed in this paper can accurately estimate road roughness using only two vehicle response quantities: the sprung mass acceleration and the unsprung mass acceleration. Additionally, it can provide a feedforward method for subsequent active suspension control.

However, this paper still has some limitations. The nonlinear suspension model considered is relatively simplistic, and in reality, the damping and stiffness of nonlinear suspensions exhibit more complex mathematical relationships, which may introduce some estimation errors. Future work should focus on improving this by adopting more accurate mathematical models. Additionally, the NARX neural network in this study was trained using simulation data, which can lead to overfitting issues when dealing with unknown road surfaces; thus, researchers should consider using actual vehicle response data for training in future efforts. Finally, in the modeling of nonlinear suspensions, a more comprehensive multi-degree-of-freedom vehicle model could be considered to enhance the estimation capability of the NARX-PASCKF under non-stationary conditions.

## Figures and Tables

**Table 1:** Classification standards of road roughness.

Road Roughness Level		$G_{q}(n_{0})/10^{-6}\;m^{3}$	
	Lower Limit	Geometric Mean	Upper Limit
A	8	16	32
B	32	64	128
C	128	256	512
D	512	1024	2048
E	2048	4096	8192

**Table 2:** Parameters of the nonlinear model.

Parameters	Symbol	Value
Sprung mass	$m_{s}$	400 kg
Unsprung mass	$m_{u}$	70 kg
Spring stiffness linear coefficient	$k_{s}^{l}$	15,680 N/m
Spring stiffness nonlinear coefficient	$k_{s}^{n}$	1568 N/m^3^
Linear damping coefficient	$c_{s}^{l}$	1780 N·s/m
Nonlinear damping coefficient	$c_{s}^{n}$	100 N·(s/m)^2^
Damping asymmetry correlation coefficient	$c_{s}^{a}$	1000 N·s/m

**Table 3:** Vehicle response process covariance.

Vehicle Response Covariance Variable	$z_{s}$	${\dot{z}}_{s}$	$z_{u}$	${\dot{z}}_{u}$
A-class	2.19 × 10^−7^	1.89 × 10^−4^	1.98 × 10^−5^	0.0021
B-class	8.89 × 10^−7^	7.76 × 10^−4^	7.79 × 10^−5^	0.0087
C-class	3.58 × 10^−6^	3.2 × 10^−3^	3.18 × 10^−4^	0.036
D-class	8.78 × 10^−6^	0.0129	1.3 × 10^−3^	0.1447
E-class	5.8 × 10^−5^	0.0522	5.1 × 10^−3^	0.6026

**Table 4:** The RMSE, MAE, and R-square comparison on a C-class road.

Methods	RMSE	MAE	R-Square
BP network	0.0056	0.0053	0.7628
NARX network	0.0030	0.0030	0.9658
Optimal ratio	46.42%	43.39%	26.61%

**Table 5:** The RMSE, MAE, and R-square comparison on a C-class road.

Methods	RMSE	MAE	R-Square
SCKF	0.0062	0.0074	0.8563
NARX-PASCKF	0.0043	0.0056	0.9108
Optimal ratio	30.64%	24.7%	6.3%

**Table 6:** The RMSE, MAE, and R-square comparison on a joint road.

Methods	RMSE	MAE	R-Square
SCKF	0.0041	0.0053	0.8935
NARX-SCKF	0.0028	0.0031	0.9743
Optimal ratio	31.7%	41.5%	9.04%

**Table 7:** The RMSE, MAE, and R-square comparison on a joint road.

Methods	RMSE	MAE	R-Square
SCKF	0.0053	0.0064	0.8689
NARX-PASCKF	0.0042	0.0047	0.9133
Optimal ratio	20.75%	26.56%	5.1%

## Data Availability

Restrictions apply to the datasets. The datasets presented in this article are not readily available because the data are part of an ongoing study or due to technical limitations. The data are not publicly available due to laboratory confidentiality regulations.
